# Family Caregiver Mental Health: Linking Family Care Regime, Intersectionality, and Stress Process Frameworks

**DOI:** 10.1093/geroni/igaa057.1616

**Published:** 2020-12-16

**Authors:** Sean Browning, Margaret Penning

**Affiliations:** University of Victoria, Victoria, British Columbia, Canada

## Abstract

Although the implications of family care regime, social location, and stress process factors for the mental health of family caregivers have been well-documented individually, there is a lack of research that integrates these factors. Yet, linking family care regime and intersectionality approaches to stress process theorizing provides us with one possible explanation of the mechanisms potentially linking family care regime and intersecting structural inequalities to mental health outcomes. This paper draws on pooled data from the 2012 and 2016 European Quality of Life Surveys (EQLS - N=6,007) to assess direct and indirect associations between family care regime and the self-reported mental health (SRMH) of family caregivers, together with the additive and interactive associations involving social location (gender, age, socio-economic status, and marital status), and stress process factors (stressors and resources). The results of a series of weighted least squares regression analyses reveal that family care regime has a direct association with SRMH and that social location and stress process factors partially mediate this association. Additionally, the results suggest that additive and interactive social location factors have direct associations with SRMH and that stress process factors also partially mediate the association. Lastly, stress process factors are associated with SRMH as expected. Overall, our findings provide initial support for the value of linking family care regime, intersectionality and stress process frameworks for an understanding of the mental health implications of family caregiving.

